# Posterior theta activity reveals an early signal of self-face recognition

**DOI:** 10.1038/s41598-023-41071-y

**Published:** 2023-08-24

**Authors:** Ilona Kotlewska, Bartłomiej Panek, Anna Nowicka, Dariusz Asanowicz

**Affiliations:** 1 Institute of Psychology, Jagiellonian University, Ingardena 6, 30-060 Krakow, Poland; 2 Laboratory of Language Neurobiology, Nencki Institute of Experimental Biology, Pasteura 3, 02-093 Warsaw, Poland

**Keywords:** Human behaviour, Attention, Perception

## Abstract

Self-related visual information, especially one’s own face and name, are processed in a specific, prioritized way. However, the spatio-temporal brain dynamics of self-prioritization have remained elusive. Moreover, it has been unclear whether this prioritization is an effect of enhancement and amplification, or rather a facilitating automatization of processing self-referential information. In this EEG study, 25 married women (who changed their surnames after marriage, so that their past and present surnames could be used as stimuli) performed a detection task with faces and names from five categories: self, self from the past, friend, famous, and unknown person. The aim was to determine the temporal and spatial characteristics of early electrophysiological markers of self-referential processing. We report results of event-related component (ERP) and time–frequency analyses. In the ERPs, the earliest self-relevance effect was displayed only 300 ms after stimulus onset in the midfrontal N2, and later in the parietal P3b, independently of the stimulus type. No self-relevance effect was found on the N170 component. However, local theta power at the occipito-temporal (visual) areas and inter-regional theta phase coherence between the visual and midfrontal areas showed that self-relevance differentiation of faces began already about 100–300 ms after stimulus onset. No such early effects were found for names. The results are discussed in terms of the time-course, functional localization, stimulus-specificity, and automatization of self-prioritization.

## Introduction

Everyone knows from their own experience that self-related information has a sort of special priority. The effects of self-prioritization have been observed in various contexts and situations. Hearing one’s own name or seeing one’s own face capture and hold attention^1–4^; it is difficult to ignore personally relevant stimuli^5–7^, and self-related information or traits are remembered better^8–13^. Regardless of the functional specificity of those effects, the prioritization of self-related information is usually assumed to be quick and automatic^14–16^. For instance, looking at one's own name has been claimed to capture one’s attention automatically, unintentionally, and preconsciously^1^. This seems to imply that: (a) self-prioritization occurs early in the time-course of processing self-related information, thus its effects should be observed in modulations of early components of the electroencephalogram (EEG); (b) self-prioritization occurs already at the perceptual levels of processing, thus its effects should be observed already in the activity of sensory areas. Furthermore, it is often assumed that self-prioritization entails enhancement and amplification of processing self-related information^17–19^. However, neither of the above has been unequivocally supported by available empirical evidence. In this study, we attempt to address those issues in terms of visual perception of the most self-related stimuli: our own face and our own name.

### When does self-prioritization occur?

Several studies observed the first differentiation between self- and other-faces at about 170 ms after stimulus onset. Self-faces evoked a larger amplitude of the N170 component of the event-related potential (ERP)^20–24^. The N170 is considered as the earliest marker of formation and activation of structural face representations^25,26^, thus the results would suggest that self-prioritization begins already along with this first stage of face processing. However, no self-face effect on the N170 component was reported even more often^27–37^. It is worth noticing that the effects on the N170 were more frequently observed for familiar faces than for the self faces, but according to a recent review by Caharel and Rossion^38^, even this familiarity effect was found only in about half of the analyzed N170 studies.

In contrast, a number of EEG studies showed the earliest difference between self and other faces only at about 250 ms after stimulus onset. It was usually observed as a negative deflection of the ERP over the inferior temporal sites, called N250^30,31,33,36,39^. This N250 effect is assumed to reflect activation of the face recognition units^40^, and is sensitive to identity-specific representations of known faces^41^. The effect of N250 for self- and familiar specificity was found also for names^42^ and owned objects^43^. The latency of about 250 ms may be considered as relatively late effect—when compared to the results showing the first effects of face-specific neural processing at about 100 ms^44–47^, and evidence for categorical perception of facial emotional expressions occurring as early as 90–140 ms after stimulus onset^48–50^. Some studies suggest the N250 depends on the level of familiarity, therefore reaches the highest amplitude for self-face^27,36^. The available results are, however, not entirely consistent with the assumption of a quick and early, presumably pre-conscious, self-prioritization.

### Where does an early self-prioritization occur?

In several studies on the visual self-relevance, the first ERP self-effects were found in the occipito-temporal visual areas. This includes some of the aforementioned studies showing the self-face effect on the N170^21–24^—the component localized in the fusiform face area^51,52^. Similarly, the self-face effect on the N250 has been observed at occipito-temporal or occipito-parietal sites^27,30,31,33,36,42^. On the other hand, many studies have found the first signs of the self-face discrimination outside the visual areas; either as a decrease of the midfrontal N2^35,53^ or centro-temporal P2^28^ (for effects in occipito-temporal P2 see^54^; and frontal P2 see^27^), or as an increase of the parietal P3b^3,29,30,34,36,55–57^. Finally, several studies have found the first effects of self-prioritization occurring at both sensory and frontal areas^21–23^. In conclusion, the available literature seems to show three patterns of the functional anatomy of the early effects of self-prioritization: either (i) self-related processing is facilitated already in the sensory (visual) areas, or (ii) it is prioritized over fronto-central areas, or (iii) both. It remains unclear what factors could explain this discrepancy. Given the discrepant evidence, we cannot draw any firm conclusion on the spatio-temporal dynamics of the identification and processing of self-related sensory information.

### Is it amplification or automatization?

Another issue concerns the nature of the self-prioritization, which is most often accounted as an enhancement or amplification of the self-related information processing. A number of studies have shown that amplitude of the P3b component (centro-parietal P300) of the ERP increases in response to self-relevance, irrespectively to stimulus type. Such P3b effect was observed for self-faces^3,29,30,34,36,37,55–59^, as well as for visually presented own name^3,56,60–62^ or online nickname^63^, autobiographical information^18^, and trait adjectives evaluation^64,65^. The self-related increase of the P3b has usually been interpreted as an enhancement of neural processing, presumably related to increased allocation of attention. However, such interpretation may be questioned. First of all, there is still an ongoing debate on the functional role of the P3b component^66–70^. Secondly, an increase of the P3b was also observed along with a decrease in attentional demands (see e.g., Refs.^71–75^), which shows that the P3b can be decreased when an enhanced involvement of task-related processes is entailed by increased task demands.

Less ambiguous for interpretation are the aforementioned self-related modulations of the N170, N250, and N2. More ambiguous, however, seem to be the actual results. While in some studies the N170 and N250 were found to be larger in response to self-face or self-name^21,22,24,33,42,76^; others reported a decrease of the N250 in response to self-relevance^27,30,31,36,43^. Some studies showed significant self-related enhancement of the N170 and at the same time self-related decrease of the P2^21,23^, while others did not find the effect in the N170 yet still showed the P2 decrease at occipito-temporal sites^27,54^. The midfrontal N2, in turn, was found to have the largest amplitude in response to written own name^60^. However, a decrease of the midfrontal N2 amplitude was observed in response to self-faces, compared to other, familiar and unfamiliar faces^23,34,35^. The N2 amplitude was also decreased when arbitrary faces were assigned and recognized as own^53^. Thus, the self-related effects of the N2 are even less conforming to the enhancement hypothesis.

Larger amplitudes of the visual-evoked potentials are usually interpreted as an increased involvement of the processes reflected in these ERPs (e.g., Ref.^77^). An increase of the midfrontal N2 often reflects a stronger involvement of some forms of executive control^78^. It is therefore plausible that the N2 decrease reflects a reduced need for control in terms of e.g., allocation of attentional resources^27,53^ onto processing self-related information, or accessing self-related memories^33^. Taken together, the findings of decreased ERP amplitudes in the self-referential conditions suggest that, at least in some cases, self-prioritization may not entail enhancement and amplification. Rather, it may be an effect of perceptual facilitation attained through a less effortful and more automatic processing of self-related information. To complicate the issue even further, self-prioritization might involve both the enhancement and automatization; the two effects could occur at different stages or levels of processing.

### Are the effects of self-prioritization stimulus-specific?

The effects of early self-prioritization may be either stimulus-specific or independent of the information type. Faces are biologically significant and ecologically-salient stimuli^79^, whereas names/words are learned symbols. Face-specific processing begins by activating the ventral fusiform face area (FFA), predominantly in the right hemisphere, whereas written words—the occipito-temporal visual word form area (VWFA) in the left hemisphere. The structural characteristics and complexity of faces and words differ, thus, their processing may involve amplification and automatization at different stages and levels of processing^80–83^. The available results show that all three discussed above issues related to self-prioritization—the time-course, functional localization, and character of the effects (amplification or facilitation)—may differ significantly depending on the stimulus type. However, since the results are not consistent, it remains unclear whether—and if so, then how exactly—the self-face and self-name effects differ in terms of their time-courses and functional anatomy.

### This study

In this study, we examined self-related modulations of the EEG data recorded during a simple visual detection task in an experiment by Kotlewska and Nowicka^56^. The stimuli were faces and names from five categories: current self, self from the past, friend, famous, and unknown. Thus, self-relevance was graded from most (current self-face/name) to least (unknown face/name) self-relevant. While the earlier study^56^ was focused specifically on similarities between the present and past selves, in the current study we examined the EEG data in terms of the spatiotemporal neural dynamics underlying self-relevance differentiation at early stages of visual processing. The aim was twofold: (a) to determine general and stimulus-specific spatio-temporal changes in electrophysiological brain activity related to the increasing self-relevance; and (b) to interpret and discuss the findings in relation to the four issues introduced above: the time-course, functional localization, nature (amplification or facilitation), and stimulus-specificity of the self-prioritization effects.

Following the previous studies reporting the effects of self-face and self-name processing, we analyzed the N170, N2, and P3b components of the ERPs. However, our main focus was on the local and inter-regional time–frequency activity in the theta-band. While the ERP analysis is an essential tool for investigating the time-course of stimulus-related processes, it reveals only a fraction of the activity of interest. The time–frequency analysis allows to detect band-specific spectral dynamics of local brain activity as well as of long-distance functional connectivity^84,85^. As the ERPs do not give a coherent picture of precise spatio-temporal characteristics of self-prioritization, the oscillatory activity may reveal some missing parts of the puzzle. For instance, a stronger theta activity over midfrontal areas was reported for familiar and famous faces than unknown ones^86^, and for self-relevant personally familiar face (participants’ grandmother) than for unknown face^87^. Theta oscillations were also found to differentiate upright and inverted faces and bodies^88^. Also, while theta activity did not differentiate spoken own-name when it was task-irrelevant^89,90^, a larger left-lateralized theta synchronization was observed when the own-name was task-relevant^90^. Most importantly, Miyakoshi and colleagues^31^ showed a smaller increase of local theta-band phase coherence in the fusiform area in response to self-face.

## Methods

### Participants

Twenty-five females participated in the study (mean age 35.7; SD 8.7). All participants had normal or corrected-to-normal vision, reported normal color vision and no history of neurological disorders. All were paid for their participation. Only married woman who have changed their surnames after marriage were recruited, so that their current and past surnames could be used in the 'present self' and 'past self' task conditions, respectively. The required minimal length of marriage was three years, and it varied from 3 to 37 years (mean 9.64; SD 8.86). The experimental protocol was approved by the Human Ethics Committee of the SWPS University of Social Sciences and Humanities (Warsaw, Poland). The study was carried out in accordance with the guidelines and regulations of the Declaration of Helsinki, and written informed consent was obtained from each participant before the experiment.

### Stimuli and procedure

Participants performed a simple visual detection task. One stimulus was presented in each trial of the task and participants responded by pressing a dedicated response button regardless of the stimulus type. The stimuli were faces and names from five categories: (1) present participants' marital names and current photos of their faces, (2) participants' family names and photos of their faces from the past, (3) names and faces of participants' close friends, (4) names and faces of famous persons (celebrities), and (5) names and faces of persons unknown to participants. Before the experiment, participants delivered photographs presenting their current face, photo of their face taken just before marriage, and the current photo of a female close friend. One restriction was made on the choice of the friend: first and last names (past or present) of the participant and the friend had to differ. All names were of Polish origin. Mean length of names was 15 letters. The length did not differ significantly between the categories (for details see^56^). The stimulus set was tailored individually for each participant. Famous and unknown names were selected to match the length of first and last name, and faces of famous and unknown persons (downloaded from the Internet) were matched to the age and appearance of the participants. Before the experiment, each participant confirmed whether she was familiar with the famous and unknown people selected for their stimulus set.

Pictures of faces were rendered grayscale. The faces were extracted from their backgrounds. Picture luminance was matched to the color statistics of a single picture. One image of face was shown per category. In the name condition, stimuli consisted of first and last names (further referred to as “names”), and were written with white letters. The stimuli were presented on a black background. The stimulus size ranged from about 2 × 3° to 2 × 6° for names, and 5 × 4° to 5 × 6° for faces. The stimuli were displayed centrally, at fixation, on a 19’ computer monitor.

Participants were asked to respond to each stimulus as quickly as possible by pressing always the same button on a response pad (Cedrus, San Pedro, CA, USA). The instructions were introduced twice: first verbally by the experimenter and then displayed on the monitor before the experiment started. Subjects had to confirm their understanding of the instruction by pressing the appropriate button.

**Figure 1:**
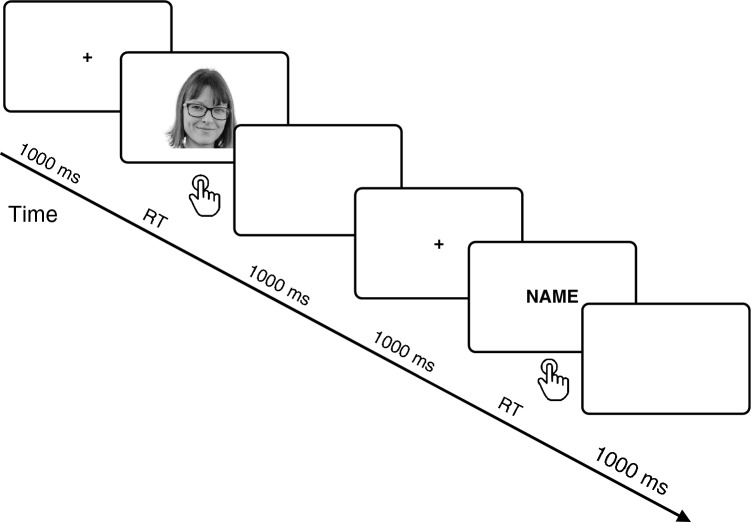
An example of stimuli and sequence of events in a trial of the stimulus detection task. (The example image of self-face is a photograph of one of the co-authors).

The stimulus presentation procedure is shown in Fig. 1 (the example picture is a photograph of one of the authors). Each trial consisted of the following sequence of events: a blank screen displayed for 1 s, a fixation cross (white cross) displayed for 1 s, and the target stimulus displayed until the response. A new trial began automatically after the response, or after 3 s if the participant did not respond. The stimulus sequence was pseudo-randomized so that no more than three stimuli of the same type or the same condition were presented consecutively. Stimulus of each type and category was repeated 30 times. The task lasted approximately 15 min. The task was performed in an acoustically and electrically shielded room.

### EEG recording and preprocessing

EEG was recorded from 62 scalp sites using Brain Products Quick Amp amplifier and BrainVision Recorder^®^ software (Brain Products, Gilching, Germany). Ag–AgCl electrodes were mounted on an elastic cap (ActiCAP, Munich, Germany) and positioned according to the extended 10–20 system. Electrode impedance was kept below 5 kΩ. The EEG was recorded against an average of all channels calculated by the amplifier hardware. The sampling rate was 500 Hz. Off-line EEG data preprocessing was done using BrainVision Analyzer^®^ software (version 2, Brain Products, Gilching, Germany). Data were re-referenced to the mean signal from earlobes, filtered with a 0.1–80 Hz band-pass and a 50 Hz band-rejection filter (Butterworth zero-phase filters, attenuation of 12 dB/octave), and split into segments beginning at− 800 ms and ending at 1000 ms relative to target onset. Segmented data were baselined to the first 200 ms before target onset (i.e., − 200 to 0 ms). Ocular artifacts were corrected by means of independent component analysis (ICA). One ICA component representing blinks was rejected per participant. Next, after resetting the baseline, the Analyzer’s semi-automatic artifact rejection method was applied. The maximum permitted voltage step per sampling point was 50 μV, the maximum permitted absolute difference between two values in the segment was 200 μV, and the lowest permitted activity in the 100 ms interval was 0.5 μV. The overall average of segments that passed the artifact rejection was 98% (range 94–100%) The averages of accepted segments in the name and face conditions, respectively, were: 98 and 97% for present self, 98 and 97% for past self, 98 and 97% for friend, 97 and 97% for famous, 97 and 97% for unknown.

### Event-related potential (ERP) analysis

**Figure 2:**
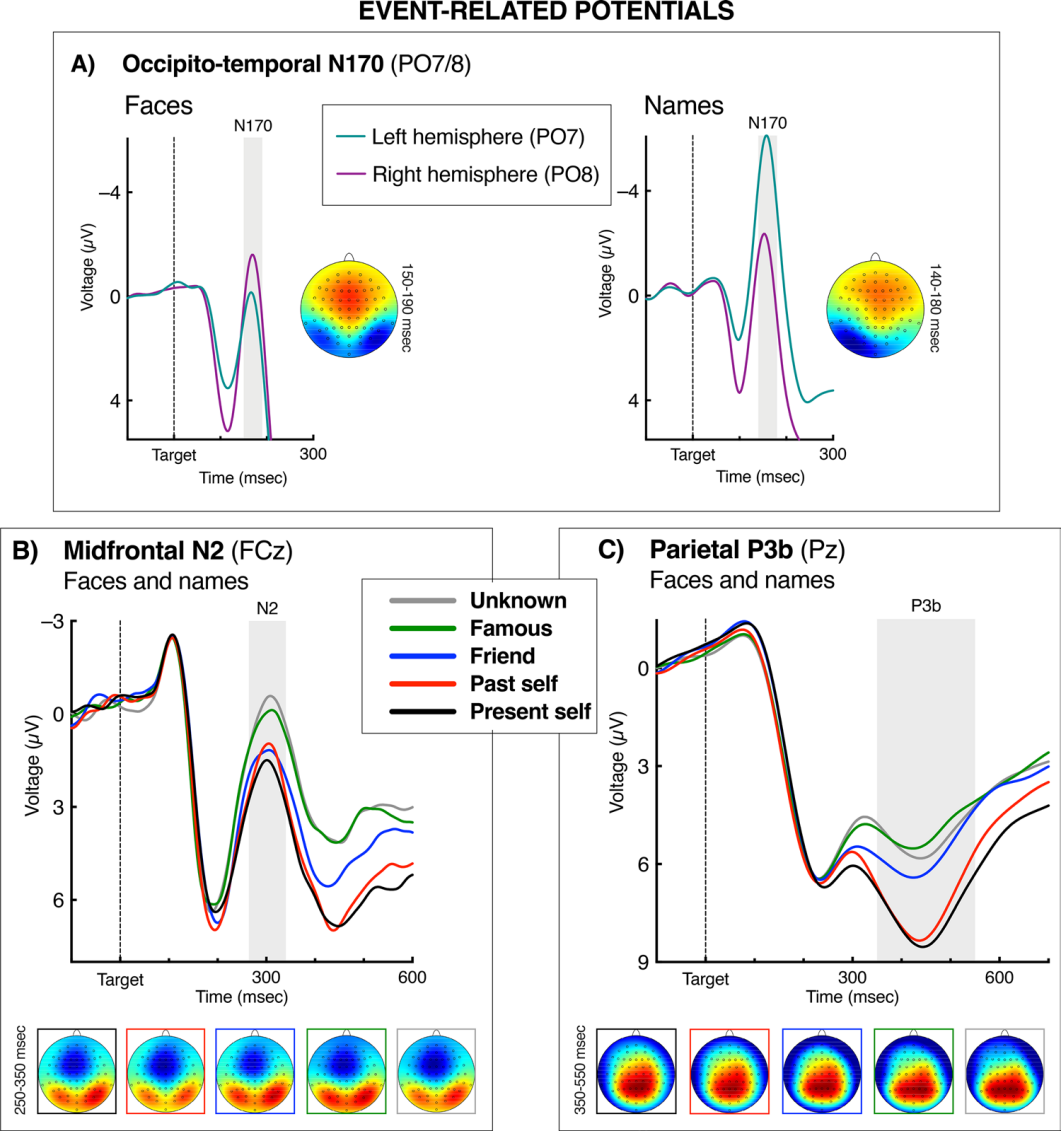
Event-related potentials (ERPs). Panel (**A**) shows grand averages of visual evoked potentials (VEPs) from occipito-temporal areas in the left and right hemisphere (PO7 and PO8) for faces (left panel) and names (right panel). The head maps show topographies of the N170 component. The data are averaged across the Self Relevance conditions because the effects of this factor on N170 amplitudes were not significant. The depicted N1 results show typical hemispheric lateralization of face and name processing. Panels (**B**) and (**C**) show grand averages of the ERPs from FCz and Pz, respectively. The data are averaged across the Stimulus Type conditions because Self Relevance effects did not differ significantly between faces and names. The head maps show topographies of the midfrontal N2 (**B**) and centro-parietal P3b (**C**) for the five Self Relevance conditions, also averaged across the Stimulus Type conditions. Colors of frames denote the conditions of Self Relevance. The N2 topography data were high-pass filtered above 2 Hz to remove the slow parietal positivity that otherwise would mask the N2 peak. The depicted results show that the stimulus self relevance decreased the N2 amplitudes and increased the P3b amplitudes. Stimulus Type did not interact with these Self Relevance effects. For all panels: Negative voltage points upward. Time-point zero is stimulus onset. The gray areas indicate the time windows in which the ERP components were measured for statistical analysis. The head maps depict topographies of the ERP components selected by the gray areas. The maps are min–max scaled, with positive polarity in red, negative polarity in blue. The head view is from above.

Before the ERP analysis, artifact-free segments were averaged over each experimental condition per participant. The ERPs of interest—N170, midfrontal N2, and P3b—have all been well-established in the literature, both empirically and theoretically, thus the electrode sites for the ERP analysis were selected a priori. Data inspection confirmed that the ERPs had the expected topographies with amplitude peaks at the selected sites (see Fig. 2). The ERP time-windows were determined using a collapsed localizer approach. i.e., on the waveforms averaged across the relevant experimental conditions (cf. Ref.^91^). All the analyzed ERPs formed regular waveforms with well-defined peaks within the selected time-windows (see Fig. 2). The amplitudes of the ERPs were measured as a mean amplitude in a given time-window. The amplitudes of the visual-evoked N170 component were measured from the occipito-temporal sites PO7 and PO8 (cf. Ref.^92^). The latencies of face-evoked N170 were about 10 ms longer than of the name-evoked N170 (see Fig. 2), thus, to avoid any between-condition artifacts of mismatch between the peaks, we measured the N170 in a 150–190 ms window for faces, and 140–180 ms for names. (We compared the obtained statistical results with the analysis of one time-window for each condition, 140–190 ms, and found the same pattern of effects.) The midfrontal N2 component was measured at FCz (cf. Ref.^23^) as mean amplitude 250–350 ms after target onset, relative to its preceding P2 component measured within 180–220 ms (the N2 was assessed peak-to-peak to nullify potential impact of between-condition variance in the preceding P2, cf. Refs.^93,94^). The P3b component was measured at Pz (cf. Ref.^67^) as mean voltage 350–550 ms after target onset.

### EEG power and phase decomposition

The segmented artifact-free data was decomposed into time–frequency representations using custom-written MATLAB (The MathWorks, Natick, MA, USA) scripts based on published code^84,95^. To obtain power and phase values, the single-trial data was convoluted via complex Morlet wavelets defined as complex sine waves tapered by a Gaussian. The power spectrum of the EEG was multiplied by the spectrum of complex Morlet wavelets with the frequencies of the wavelets increased logarithmically from 1 to 40 Hz in 40 steps. The number of wavelet cycles in the Gaussian that tapers the sine waves was increased logarithmically from 3 to 8. This allowed to obtain an optimal tradeoff between temporal and frequency resolutions^96^.

The obtained power values (µV^2^) were baseline-corrected as a percentage change relative to the pre-stimulus baseline at each frequency band. The baseline was computed as the average power across all experimental conditions, from − 400 to − 100 ms before target onset. For statistical evaluations of target-related theta modulations, power values from relevant sites (PO7 and PO8 for analysis of visual activity; FCz, Fz, and AFz for analysis of midfrontal activity) were averaged over a time–frequency window of 100–300 ms and 5–7 Hz. This theta window was specified based on previous studies and confirmed after inspection of the data. More detailed descriptions of the time–frequency decomposition method (including the algorithm details) can be found in our previous articles^72,73,94^.

### Multivariate spatio-spectral decomposition

To estimate the global topology of the task-related theta activity, we used the method of generalized eigenvalue decomposition (GED^97–99^; also referred to as joint decorrelation^100^). The GED is a feature-guided multivariate source separation technique that contrasts and maximally separates two covariance matrices of two a priori-specified data-features of interest. The first matrix is the channel (electrode) covariance of the relevant signal. Because our aim here was to isolate theta activity, the signal matrix (S) was derived from the data narrow band-filtered in the theta band^98,101^. The filter was centered at 5 Hz, and the range of the Gaussian spectral full-width at half-maximum (FWHM) was 3 Hz. The second matrix is the channel covariance of the reference data. Thus, our reference matrix (R) was derived from the broadband (unfiltered) EEG. Both matrices were computed for the entire post-target epoch. The covariance matrices were calculated for each participant, separately for the face and name conditions. To avoid potential stimulus-evoked transient artifacts, the phase-locked part of the signal was removed from the single-trial data prior to the GED analyses^102^. Next, the GED was applied to the matrices, which yielded a set of 62 spatial components (each being a weighted combination of all 62 channels) of the theta-band signal for each subject per condition. The components are specified by their eigenvalues that show the S/R ratio indicating the importance of each component, and eigenvectors that provide the parameters of the spatial filters, i.e., the sensor weights constituting each spatial component.

In the next step, we determined which of the obtained components were significantly contributing to the overall stimulus-related theta signal^97^. To this end, we used a non-parametric permutation testing method, which was a modified version of a parallel analysis method^101^. According to Hayton et al.^103^, parallel analysis is one of the most accurate methods of factor retention in exploratory factor analysis. Zuure et al.^101^ utilized this method for thresholding the GED components. First, theta-filtered and broadband signals were randomly assigned to the S and R matrices and submitted to the GED, which generated a random distribution of eigenvalues. One thousand iterations of the randomization were performed, and 62 eigenvalues extracted from the randomly swapped data at each iteration. After all iterations, the eigenvalues above the 95th percentile of the explained variance were selected from the full set of eigenvalues (62 × 1000) to create a random dataset (a null hypothesis distribution). This was done for each subject. The resulting datasets were averaged to obtain the group-level distribution, and compared against the eigenvalues of theta components obtained from the GED. Any component with eigenvalue greater than the maximal eigenvalue generated for the random distribution was considered significant. At the group level, three components with the highest eigenvalues exceeded the retention threshold (α = 0.05) and were selected for further analysis.

The obtained eigenvectors were used to construct spatial topographies of the three significant theta components. The component spatial representations were computed as the eigenvector multiplied by the S covariance matrix^104^. It is assumed here that the component topographies represent the underlying EEG sources projecting the signal onto the electrodes^97^. (Note however that the GED components do not indicate neural sources in the sense of delineating the exact functional anatomy.) Lastly, for further inspection, component-specific time-series data (the component eigenvector multiplied by the single-subject EEG signal) was decomposed into its time–frequency representation through convolution with complex Morlet wavelets, as previously described for sensor-data analysis.

### Inter-site phase coherence (ISPC)

To estimate theta-band connectivity between the midfrontal and visual areas (indicated in the GED analysis), the obtained phase angles (derived from the complex wavelet convolution analysis, as described above) were used to compute inter-site phase coherence (ISPC)^105,106^. (This measure is also referred to as: inter-site phase connectivity, inter-site phase clustering, inter-channel phase synchrony or ICPS, and inter-site phase-locking value or PLV.) Before the phase decomposition for ISPC analysis, the surface Laplacian filter^107,108^ (also called current source density or current scalp density) was applied to the single-trial data. We used a 10th-order Legendre polynomial, and lambda was set at 1e−5. The Laplacian is a spatial filtering method that increases spatial selectivity and attenuates volume conduction confounds, allowing effective assessments of long-distance phase-based connectivity^107,109^. ISPC estimates the consistency between band-specific phase angle values at two sites or areas of activity. It is computed as the length of the mean vector of differences between a distribution of phase angle differences over trials, at each time–frequency point. The ISPC value varies from 0 to 1, where 0 indicates no phase synchrony between two sites, i.e., randomly distributed phases, and 1 indicates identical phase values at two sites, i.e., a perfect inter-site phase coherence. The obtained ISPC values were baseline-corrected as a percentage change relative to the pre-stimulus baseline, using the same method and the same baseline time-window as for power analysis. For statistical analysis, ISPC between the FCz site (used as “seed”) and the posterior PO7 and PO8 sites was averaged over a time–frequency window of 50–400 ms and 4–8 Hz—specified after inspection of the condition-averaged time–frequency results.

### Inferential statistics

To determine both main effects and interactions between the experimental factors, all depended variables were analyzed by means of repeated-measures ANOVAs. The behavioral data were submitted into a 2 × 5 repeated measures ANOVA with Stimulus Type (faces, names) and Self Relevance (present self, past self, friend, famous, unknown) as within-subject factors. The N2 and P3b components of the ERP were analyzed using the same ANOVA design as for the behavioral data. The N170 component and theta-band activity (both occipito-temporal local power and ISPC) were analyzed using a 2 × 5 × 2 repeated measures ANOVA with Stimulus Type (faces, names), Self Relevance (present self, past self, friend, famous, unknown), and Hemisphere (left, right) as within-subject factors. When the Stimulus Type × Self Relevance interaction was significant, further analyses of Self Relevance effects were carried out separately for faces and names. The Greenhouse–Geisser correction was applied when the factor Self Relevance had more than one degree of freedom in the numerator to adjust for violations of sphericity. When the main effect of Self Relevance was significant, pairwise comparisons between the Self Relevance conditions were performed. The Bonferroni correction for multiple comparisons was applied to all pairwise tests. Statistical analyses were done in IBM SPSS Statistics 28.

## Results

The RT, P2, and P3b results from this experiment have been reported previously in Kotlewska and Nowicka^56^. The data were reprocessed and reanalyzed for the purpose of this study.

### Behavioral results

The overall mean RT was 408 ms (SD = 257 ms). Stimulus Type and Self Relevance did not affect the RTs, *F* ≤ 2.00, *p* ≥ 0.10.

### Event-related potentials (ERPs)

#### Occipito-temporal N170

Figure 2A depicts grand averages of the ERPs from the PO7 and PO8 sites, separately for the two Stimulus Type conditions. The figure shows an opposite hemispheric lateralization of the N170 amplitudes for faces (left panel) and names (right panel), indicated by the interaction between Stimulus Type and Hemisphere, *F*_1,24_ = 42.68, *p* < 0.001, *η*_*p*_^2^ = 0.64. As expected, names evoked a larger N170 in the left hemisphere (PO7) than in the right hemisphere (PO8), *F*_1,24_ = 29.02, *p* < 0.001, *η*_*p*_^2^ = 0.55; whereas the N170 evoked by faces tended to be larger in the right hemisphere than in the left, *F*_1,24_ = 4.14, *p* = 0.053, *η*_*p*_^2^ = 0.15. Importantly, Self Relevance did not significantly affect the N170 amplitudes. In the face condition, the main effect of Self Relevance did not reach the level of significance, *F*_4,96_ = 2.23, *p* = 0.084, *η*_*p*_^2^ = 0.09 (Self × Hemisphere: *F* < 1.0, n.s.), and the pairwise comparisons showed no significant differences between the five Self categories, *p*’s ≥ 0.35. In the name condition, the Self Relevance effect was far from being significant, *F* < 1.0, n.s. The Self × Stimulus Type interaction was also not significant *F* < 1.0.

#### Midfrontal N2

Grand-average ERP waveforms from FCz are shown in Fig. 2B. The N2 peaked around 300 ms after stimulus onset, and was larger for faces than names, *F*_1,24_ = 12.54, *p* = 0.002, *η*_*p*_^2^ = 0.34. Importantly, the main effect of Self Relevance was significant, *F*_4,96_ = 5.92, *p* < 0.001, *η*_*p*_^2^ = 0.20. Moreover, the Stimulus Type × Self Relevance interaction was not significant, *F*_4,96_ = 1.97, *p* = 0.12, *η*_*p*_^2^ = 0.08, indicating that the Self Relevance effect on the midfrontal N2 did not differ significantly between faces and names. The N2 amplitudes increased from smallest for present self, to larger for past self, intermediate for friend, and to largest for the famous and unknown faces; linear trend: *F*_1,24_ = 27.97 *p* < 0.001, *η*_*p*_^2^ = 0.54. Pairwise comparisons showed significant differences between present self and famous, *p* = 0.002, and between present self and unknown, *p* < 0.001.

We also quantified the positive peak that preceded the N2, i.e., P2 occurring at about 200 ms, and found no significant effects of Self Relevance, *F* ≤ 1.61, *p* ≥ 0.20 (cf. Ref.^56^).

#### Centro-parietal P3b

The grand-average ERPs from Pz are depicted in Fig. 2C. The P3b component peaked between 350–550 ms. Its amplitude was generally larger for faces than names, *F*_1,24_ = 37.03, *p* < 0.001, *η*_*p*_^2^ = 0.61. Importantly, Self Relevance modulated the P3b amplitude, *F*_4,96_ = 16.25, *p* < 0.001, *η*_*p*_^2^ = 0.40, in the direction opposite to the N2 effect. The P3b amplitude decreased from largest for self-relevant categories (present and past self), to intermediate for friend, and to smallest for famous and unknown categories; linear trend: *F*_1,24_ = 30.31 *p* < 0.001, *η*_*p*_^2^ = 0.56. As for the midfrontal N2, the interaction between Stimulus Type and Self Relevance was not significant*, F* = 1.0, indicating similar processing of Self Relevance in the centro-parietal area for faces and names. Pairwise comparisons showed that both the present and past self-face-evoked P3b were significantly larger than the P3b in the other three categories: self vs. friend, *p*’s ≤ 0.005, self vs. famous, *p*’s < 0.001, and self vs. unknown, *p*’s ≤ 0.002. See Table 1 for the estimated amplitudes of the ERP components for each experimental condition with standard error of the mean and confidence intervals.

**Table 1:** Estimated amplitudes (in µV) of the N170, N2, and P3b components of the ERPs for each experimental condition. *SEM:* standard error of the mean, *CI:* confidence intervals. See “Methods” for details.

Stimulus type	Self relevance	N170 (PO7)		N170 (PO8)		N2 (FCz)		P3b (Pz)	
		Mean (SEM)	95% CI	Mean (SEM)	95% CI	Mean (SEM)	95% CI	Mean (SEM)	95% CI
Face	Present self	− 0.06 (0.9)	− 1.9, 1.8	− 1.19 (0.8)	− 2.9, 0.5	− 3.4 (0.7)	− 4.8, − 2.0	5.82 (0.7)	4.4, 7.2
	Past self	1.0 (1.0)	− 1.0, 3.0	− 0.38 (1.0)	− 2.3, 1.6	− 3.61 (0.8)	− 5.3, − 2.0	5.39 (0.7)	4.0, 6.8
	Friend	− 0.01 (1.0)	− 2.0, 2.0	− 1.53 (0.9)	− 3.3, 0.2	− 3.36 (0.6)	− 4.7, − 2.0	4.25 (0.7)	2.9, 5.6
	Famous	0.36 (1.0)	− 1.8, 2.5	− 1.52 (1.0)	− 3.5, 0.5	− 3.88 (0.9)	− 5.7, − 2.0	2.85 (0.6)	1.6, 4.2
	Unknown	0.68 (0.8)	− 1.1, 2.4	− 0.25 (0.9)	− 2.1, 1.5	− 4.43 (0.7)	− 6.0, − 2.9	3.11 (0.6)	2.0, 4.3
Name	Present self	− 5.65 (1.0)	− 7.7, − 3.6	− 2.21 (1.0)	− 4.3, − 0.1	− 4.46 (1.0)	− 6.4, − 2.5	9.65 (0.9)	7.9, 11.4
	Past self	− 5.39 (0.9)	− 7.2, − 3.6	− 1.59 (0.9)	− 3.5, 0.3	− 5.97 (1.1)	− 8.2, − 3.7	9.58 (0.9)	7.7, 11.4
	Friend	− 5.54 (1.0)	− 7.5, − 3.6	− 1.67 (1.0)	− 3.7, 0.4	− 6.32 (1.0)	− 8.5, − 4.2	7.38 (0.7)	5.9, 8.8
	Famous	− 5.6 (1.0)	− 7.7, − 3.5	− 1.58 (1.0)	− 3.6, 0.4	− 6.89 (0.9)	− 8.8, − 5.0	7.23 (0.7)	5.7, 8.8
	Unknown	− 5.19 (0.9)	− 7.1, − 3.2	− 1.34 (0.9)	− 3.2, 0.5	− 7.01 (1.0)	− 9.0, − 5.0	7.51 (0.8)	5.8, 9.2

### Occipito-temporal theta power

**Figure 3:**
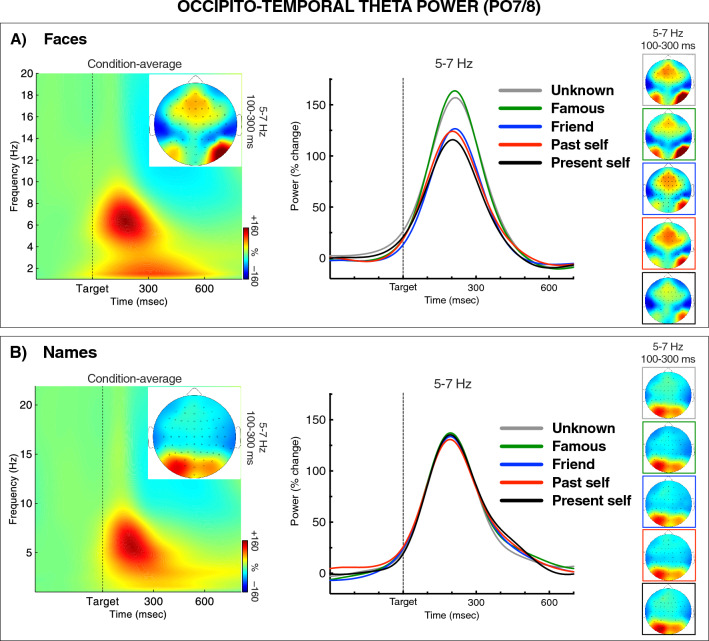
Stimulus-related modulations of EEG power at the occipito-temporal sites PO7 and PO8 in the face (**A**) and name conditions (**B**). The time–frequency plots on the left depict power spectra averaged over the Self Relevance conditions and PO7/8 sites, along with topographies for the relevant time–frequency window. The charts in the middle show grand averages of theta power over time for each task condition. The head maps on the right show topographies of theta power for the analyzed time–frequency window. The colors of lines and frames denote Self Relevance conditions. The depicted results show that the increasing self-relevance of faces, but not names, attenuated the stimulus-related increase of the occipito-temporal theta power.

Figure 3 shows EEG power from the sites PO7 and PO8, separately for faces (upper panel) and names (lower panel). The time–frequency maps (on the left) depict power spectra averaged over the PO7/8 electrodes and Self Relevance conditions, along with topographies of theta-band power. The graphs (in the middle) and head maps (on the right) show grand averages and topographies of theta power for each condition of Self Relevance.

As seen in the figure, there was a prominent burst of theta power over the occipito-temporal sites at about 100–300 ms after stimulus onset. Expectedly, as in the visual-evoked potentials, there was a significant interaction between Stimulus Type and Hemisphere, *F*_1,24_ = 21.70, *p* < 0.001, *η*_*p*_^2^ = 0.48, indicating a right-lateralization for faces, and a reversed asymmetry for names. The main effects of Stimulus Type and Hemisphere were insignificant, *F*s ≤ 1.0. Most importantly, the main effect of Self Relevance was significant, *F*_4,96_ = 4.40, *p* = 0.006, *η*_*p*_^2^ = 0.16, and so was its interaction with Stimulus Type, *F*_4,96_ = 3.78, *p* = 0.012, *η*_*p*_^2^ = 0.14. The following analysis of Self Relevance effects were therefore conducted separately for faces and names.

In the face condition, the main effect of Self Relevance was significant, *F*_4,96_ = 6.53, *p* < 0.001, *η*_*p*_^2^ = 0.21. Theta power increased from smallest for present self to largest for the famous and unknown categories. This linear trend was significant, *F*_1,24_ = 14.14, *p* < 0.001, *η*_*p*_^2^ = 0.37. The interaction of Self Relevance and Hemisphere was not significant, *F* < 1.0. Pairwise comparisons showed that present self-elicited significantly lower posterior theta power compared to famous face, *p* = 0.005, and to unknown face, *p* = 0.042. The past self differed significantly from famous face, *p* = 0.037. A marginal difference was found between friend’s face and unknown face, *p* = 0.053. No significant difference was found between the two self categories (present vs. past, n.s.).

In the name condition, none of the Self Relevance effects on posterior theta power reached the level of statistical significance (*Fs* < 1.0, n.s., see Fig. 3).

### Theta topology (GED results)

Our sensor-level results showed the most prominent theta power bursts over the lateral occipito-temporal areas. However, in the face condition, a noteworthy stimulus-related increase of theta power was present also over anterior midline sites (see the head maps in Fig. 3A). Anterior midline activity has been indicated in fMRI studies as related to processing of self-related information^10,110–113^. Hence, our next questions were (a) whether anterior theta was functionally connected with posterior theta, and (b) whether this connectivity was related to processing of the stimulus self-relevance, as was the local posterior activity. We therefore proceeded to examine source-level topology of the whole-brain theta-band activity, aiming at more precise delineating the sites that could be further examined in terms of their local activity and their inter-regional functional connectivity. To this end, we used the GED-based method of multivariate spatio-spectral decomposition (see “Methods” for details).

**Figure 4:**
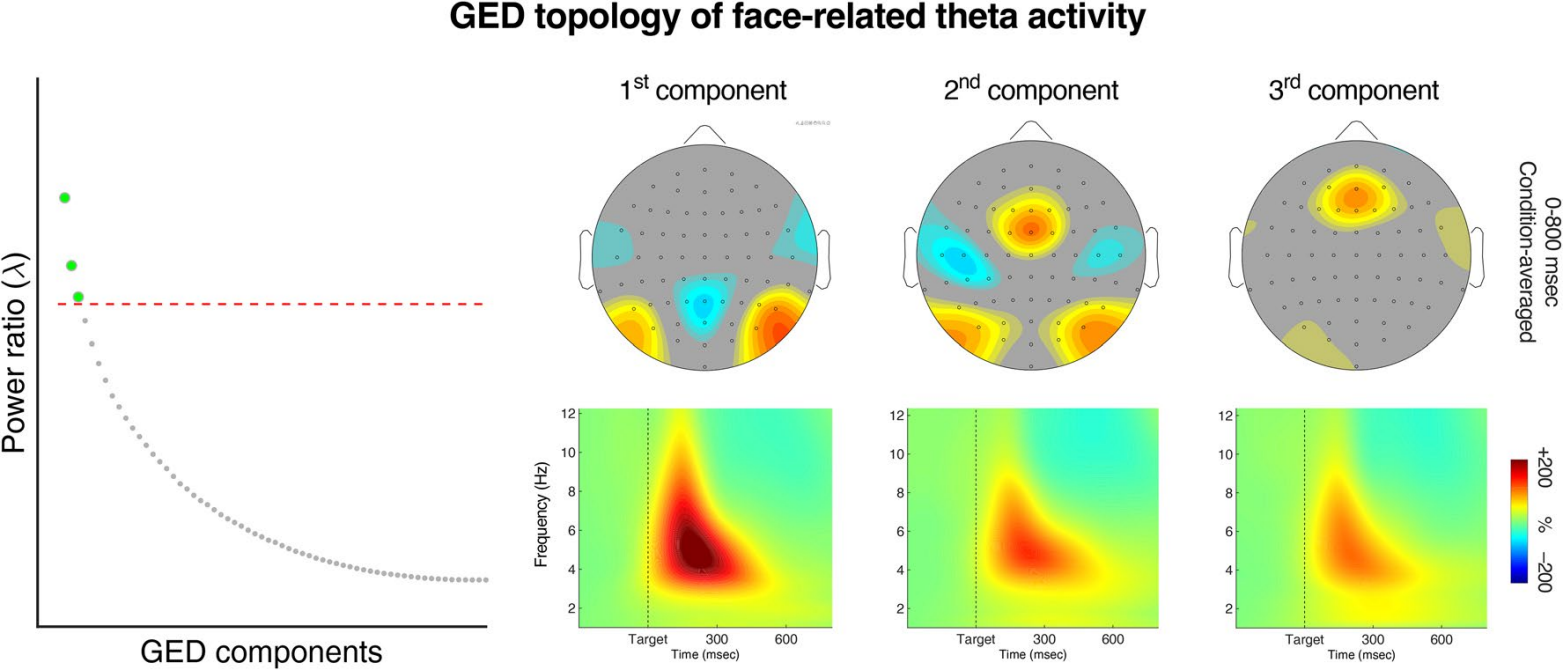
Theta topology defined by the GED. The scree plot on the left shows averaged eigenvalues of all GED components, with statistical significance cutoff determined by permutation testing. Eigenvalues indicate the importance of each component for separating the signal covariance matrix from the reference covariance matrix (i.e., S/R power ratio). The head maps show activation topographies of the three significant components of theta activity defined by GED. The plots below show time–frequency power decompositions of the three GED components, indicating homogenous activity in the theta band.

The GED results revealed three components significantly contributing to the overall stimulus-related theta-band signal (see the left panel of Fig. 4). Spatial topographies of the components averaged across participants are shown in Fig. 4, along with plots of averaged time–frequency decomposition of the components. Note that the components' power spectra showed very similar time courses of the stimulus-related modulations, indicating that the components contributed to the overall theta variance within the same time-window of about 100–400 ms after target onset.

The first component isolated the local lateral-posterior theta, which was already demonstrated as the strongest theta signal in the sensor-level analysis above. The anterior theta was in turn isolated in the third component, confirming the anterior midline theta roughly indicated on the sensor-level topography. Most interesting is the second component, which isolated again the lateral-posterior areas, but this time along with the midfrontal site. Apparently, this component was driven not by a local source, but anatomically distributed sources captured into one component.

### Inter-regional theta phase coherence (ISPC results)

The spatio-temporal activation pattern isolated in the second GED component seems to indicate a network of nodes that were parallelly recruited, and time–frequency synchronized in the theta-band (cf. Refs.^97,114^). The midfrontal activation indicated in this component presumably represents the midfrontal theta^115^, which is often interpreted as a hub of long-distance connectivity that entrains task-related functional nodes through inter-regional phase coherence in the theta band^73,116,117^. Following this interpretation, in the next step of analysis, we used the midfrontal site (FCz electrode) as a “seed” of inter-regional connectivity (cf. Refs.^73,118,119^) and examined ISPC between the midfrontal and lateral-occipital activations.

The ISPC results showed a conspicuous increase of phase coherence between the two sites (relative to baseline) from 100 to 400 ms after stimulus onset, covering the full range of theta band. The omnibus ANOVA showed two relevant interactions. The Stimulus Type × Hemisphere interaction, *F*_1,24_ = 22.06, *p* < 0.001, *η*_*p*_^2^ = 0.48, indicated again the lateralized effects for faces and names. More importantly, the Stimulus Type × Self Relevance interaction, *F*_4,96_ = 2.80, *p* = 0.038, *η*_*p*_^2^ = 0.10, indicated a significant difference between the face and name conditions in the effects of Self Relevance.

**Figure 5:**
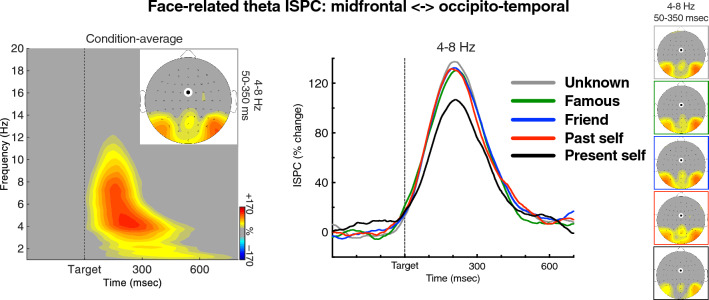
Inter-site phase coherence (ISPC) between the midfrontal and lateral-posterior areas in the face conditions. The time–frequency plot shows ISPC averaged over the Self Relevance conditions and hemispheres, along with a topographical map of theta-band ISPC for the analyzed time–frequency window. The chart in the middle shows grand averages of theta ISPC over time for the five Self Relevance conditions. The head maps on the right show topographies of theta ISPC. The colors of lines and frames denote the Self Relevance conditions. “Hotter” colors on time–frequency plot and head maps indicate more robust phase synchronization. The black on white dot on head maps denotes the midfrontal “seed”. The depicted results show that the stimulus-related increase of theta phase-synchrony between the midfrontal and posterior areas was smallest in the self-face condition.

The ISPC results for the face condition is shown in Fig. 5. The main effect of face self-relevance did not reach significance, *F*_4,96_ = 2.16, *p* = 0.094, *η*_*p*_^2^ = 0.08, but the linear trend was again significant, *F*_1,24_ = 10.39 *p* = 0.004, *η*_*p*_^2^ = 0.30, reflecting a gradual increase of the signal, from weakest for present self to strongest for self-irrelevant categories, consistently with the effects on posterior theta power. Pairwise comparisons of present self-face with the other face categories reached the alpha-unadjusted significance level (*p*’s = 0.048, 0.026, 0.007, 0.008, respectively from past self to unknown), but did not survive the Bonferroni correction (*p*’s = 0.48, 0.26, 0.071, 0.084, respectively). In conclusion, the theta ISPC results showed a trend consistent with the theta power results. In the name condition, none of the effects of Self Relevance was significant, *F*s < 1.0. Estimations of theta-band power and ISPC for each experimental condition are provided in Table 2.

**Table 2:** Estimations of theta-band power and inter-site phase coherence (ISPC) for each experimental condition (in % change). *SEM:* standard error of the mean, *CI:* confidence intervals. See “Methods” for details.

Stimulus type	Self relevance	Theta power				Theta ISPC			
		PO7		PO8		PO7-FCz		PO8-FCz	
		Mean (SEM)	95% CI	Mean (SEM)	95% CI	Mean (SEM)	95% CI	Mean (SEM)	95% CI
Face	Present self	88 (26)	35, 141	109 (19)	70, 147	67 (12)	42, 92	81 (10)	60, 102
	Past self	94 (27)	38, 150	118 (26)	65, 171	88 (14)	60, 116	95 (13)	69, 121
	Friend	98 (31)	34, 163	117 (26)	64, 170	92 (12)	67, 117	95 (12)	70, 121
	Famous	117 (30)	55, 178	161 (29)	102, 220	85 (14)	56, 114	98 (13)	72, 124
	Unknown	122 (30)	60, 185	148 (30)	87, 210	94 (13)	67, 121	97 (12)	72, 122
Name	Present self	127 (27)	71, 184	99 (18)	62, 137	72 (9)	54, 90	66 (9)	47, 85
	Past self	125 (21)	82, 167	95 (15)	64, 126	71 (9)	53, 89	52 (10)	31, 73
	Friend	127 (23)	80, 174	97 (16)	64, 130	74 (8)	57, 90	47 (9)	29, 66
	Famous	135 (27)	80, 190	93 (15)	62, 125	75 (11)	53, 97	57 (10)	35, 78
	Unknown	126 (24)	76, 176	101 (17)	67, 135	69 (9)	50, 89	41 (8)	25, 57

### Anterior midline theta power

In the last analysis step, we examined the local anterior midline theta activity, indicated in the second and third GED components. To this end, power from the anterior sites AFz, Fz, and FCz was measured at the same time–frequency window as occipital power, and entered the omnibus ANOVA (with AFz, Fz, and FCz, instead PO7 and PO8). The results showed that anterior theta power was generally larger for faces than names, *F*_1,24_ = 9.69, *p* = 0.005, *η*_*p*_^2^ = 0.29. Marginally significant was the interaction Stimulus Type × Self Relevance × Electrode, *F*_8,192_ = 2.42, *p* = 0.056, *η*_*p*_^2^ = 0.09, because in some conditions power tended to be largest at AFz. However, we found no significant differences between the self-relevance conditions, *F*s ≤ 2.09, *p* ≥ 0.12.

## Discussion

### Summary of results

#### Behavioral results

No differences in RTs between experimental conditions were found. Specifically, no RTs effects for self-referential stimuli were observed although such effects have been reported in previous studies (e.g., Ref.^3^, for review see Ref.^120^). It is worth mentioning that the task used in the current study was very simple; it did not require discriminating between stimuli, thus no in-depth processing of incoming information was required. Still, analyses of neural activity showed several significant effects (see below). It should be noted, however, that our EEG results showed self-relevance effects on amplitudes and not latencies of the stimulus-related modulations. Thus, the RT result is not incompatible with the EEG results.

#### ERP results

The amplitudes of visual-evoked N170 showed typical effects of stimulus-specific hemispheric lateralization. Faces tended to evoke larger N170 in the right hemisphere than in the left, in line with evidence for lateralization of face processing^121,122^ and particularly the asymmetry of the FFA^52,123,124^. In turn, the N170 evoked by names was larger in the left hemisphere, presumably reflecting the involvement of the visual word form area^125–127^. Importantly, self-relevance of the visual stimuli did not affect the N170.

In our ERP results, the first significant effect of self-relevance appeared only at about 300 ms after stimulus onset. The self-relevant stimuli evoked a smaller midfrontal N2 component than the stimuli from the other categories, which is in line with several previous studies^23,27,33,35,53^. The self-relevance effect was then reversed into a larger P3b amplitudes in the self-relevant conditions, also in accordance with previous studies^3,18,31,36,37,55,58,59,128^. Importantly, neither the N2 nor P3b self-referential effects differed between faces and names, thus the effects may reflect a domain-general self-prioritization.

The ERP results therefore suggest that (a) self-relevance affects stimulus processing only at later, post-perceptual stages of processing, carried out in the midfrontal and parietal areas, and (b) the self-relevance effects are independent of the type of processed information. However, our time–frequency results show that such conclusions would be premature.

#### Time–frequency results

The EEG power results showed typical stimulus-related bursts of local activity in the theta-band over visual areas in both hemispheres, emerging within 100–300 ms after stimulus onset. Importantly, the self-related faces (present and past self) elicited a smaller increase of this occipito-temporal theta power than the self-irrelevant faces (i.e., famous and unknown face). Moreover, this early effect of self-relevance was present only for faces and not for names. Unlike the ERPs, the theta power results show that self-relevance may modulate already the early perceptual processing in visual areas, and this early self-relevance effect is stimulus-specific. This result corresponds with the previous observation of lower local theta phase synchrony in the right fusiform area during seeing self-face, compared to other familiar faces^31^.

In the next step, we identified the whole-brain topology of theta-band activity using a GED-based spatial filtering method^97^. The GED results showed that the second most-prominent area of theta activity—after the posterior (visual) theta—was over the midfrontal area. We then assessed inter-site phase coherence between the midfrontal and occipito-temporal areas of activity, to estimate their functional connectivity. The ISPC results showed that there was a significant burst of stimulus-related connectivity in the theta-band between these areas. This is in line with evidence showing that midfrontal theta—typically originating from the anterior cingulate cortex (ACC) and medial frontal cortex (MFC)—functions as a “hub” of long-distance task-related connectivity, which is carried out through theta phase coherence^73,115,116^. This stimulus-related phasic increase of theta ISPC tended to be smaller in the self-face condition than in the other face conditions. No such effect was found for names. Thus, although power and phase are generally independent of each other^84^, our results of theta occipital power and ISPC are consistent in showing the smallest stimulus-related modulations for self-face, and no self-relevance effects for names.

### The time-course and functional anatomy of self-effects in visual perception

The first two questions we have asked were: how early in the processing chain is self-relevance differentiated from other sensory information, and where in the brain does the differentiation occur? The present findings of stimulus-related modulations of local occipito-temporal theta power suggest that self-face is differentiated within the first 100–300 ms, and the differentiation entails modulations of local activity in visual areas as well as of functional connectivity between the visual and midfrontal areas. The results conform to the previous findings showing an early local activity related to self-processing occurring parallelly in both the visual and midfrontal areas^21–23^. It also corresponds to fMRI studies showing the involvement of midfrontal areas in self-referential processing^110–112,129^, recently demonstrated also in primates^130^. As these local posterior and midfrontal areas are assumed to be part of an integrated system (e.g., a self-attention network^131^), we certainly should expect them to be functionally connected. Our ISPC results show that such inter-regional connectivity may be mechanistically carried out through a synchronization of theta-band activity, which is in line with the current accounts of long-distance neuronal communication^132–134^.

#### Stimulus-specificity of early self-relevance effect

An important aspect of the present results is that the early self-effect in the theta-band activity was found for faces and not for names. Thus, our results provide a potentially important data point contributing to understanding the specificity of face processing. The critical neural nodes involved in visual perception of faces and words—the FFA and VWFA—are anatomically located in similar areas of the fusiform gyri, in the right and left hemisphere, respectively^81^. In the present results, the assumed involvement of the two areas in processing faces and words is presumably reflected by the opposite hemispheric lateralization of the N170. Based on the functional anatomy, it would be reasonable to expect a similar time-course of self-referential differentiation of faces and names. However, the results showed the contrary. While the latency of name-evoked N170 was generally about 10 ms shorter than for face-evoked N170 (which might reflect the slightly more posterior localization of the VWFA than the FFA), the self-name differentiation was found only at about 300 ms after stimulus onset, as a modulation of the midfrontal N2 (and then P3b). The early self-differentiation of faces is plausibly based on the biologically determined mechanisms of face perception. The differentiation of self-name may rely on more time-consuming neural apparatus, because it may also need to involve later, semantic stages of processing, in addition to early visual encoding^126^.

#### The N170 discrepancy

Interestingly, as in several previous studies^27–36^, the self-face differentiation was not reflected in the N170, which suggests that this early visual ERP may not be sensitive to one’s own facial identity. In this line, it has been argued that the N170 may reflect specifically the processing of stimulus structural configurations, independently of facial expression or identity. This however contradicts with studies reporting positive N170 self-face effects^21–24^, and it is not clear what could explain the discrepancy. The problem is analogous to the issue of encoding facial emotions. While in some studies early signs of processing facial expressions were observed over the visual occipito-temporal areas^48,135,136^, others found such effects only over the fronto-central regions^137–139^. This sparked a debate on whether facial expressions are encoded along with the perceptual processing of facial structural configurations (as indexed by the N170) and what is the neural architecture of integrations between these aspects of face processing^25,126^. The same questions apply to the issue of encoding self-relevance.

One study demonstrated that the N170 could show the effects of facial expressions only because the N170 was spatially and temporally overlapping with activity from the expression-sensitive early posterior negativity, and not because the N170 per se is sensitive to facial expressions^140^. If the N170 is indeed not sensitive to face identity—as the present results seem suggest, possibly a similar overlap might occur during processing of face identity. This might explain why some studies did observe the N170 effects^21–24^. That is, the early structural processing and the identity processing might be carried out parallelly through partially overlapping but independent processes. We may speculate that the former might be expressed in the ERPs as the N170, while the latter in the modulations of occipito-temporal theta-band activity, but it obviously requires further evidence.

On the other hand, the meta-analysis by Hinojosa et al.^25^ showed that the N170 may in fact be affected by facial identity and expressions. This may therefore also explain the observations of the positive N170 self-face effects^21–24^. Still, it does not explain the discrepancy between the studies. It may therefore be that the self-face sensitivity of the N170 reveals only under specific conditions. Plausibly, specific task demands interact with and modulate the early self-referential visual processing, possibly by some top-down executive regulations of task-set parameters (see the section below on the issue of stimulus task-relevance).

Another relevant issue in this context is the neurobiological meaning of an enhanced N170. In terms of changes in the actual neural dynamics, the self-face related increase of the N170 amplitude may reflect either a stronger activation of the same neural sources or additional sources activated specifically by self-faces. Most plausibly, the N170 originates from a massive synchronized increase of postsynaptic neural activity, both time-locked and phase-locked to stimulus onset. Accordingly, the enhanced N170 (either to faces in general, or to self-face specifically) would reflect an increased recruitment of those neural sources^92^.

### Self-relevance decreases stimulus-related activity

Another question we have asked here was whether self-referential processing entails an amplification of the self-related information, as it has often been assumed. Contradictory to this assumption, our results showed that self-relevance decreased the stimulus-related effects on the N2, posterior theta power, and theta ISPC. The interpretation of these effects is relatively straightforward—they do not show an amplification, but rather a smaller involvement of the underlying process, compared to the self-unrelated stimuli. The only exception was the P3b, but, as mentioned in the introduction, its interpretation is not as straightforward. The self-related P3b amplitude increase might reflect a decrease of the involvement of the underlying process, not their amplification or enhancement. It is also unclear what exact processes are reflected by the P3b (see the “Introduction”).

The smaller stimulus-related amplitude increase of the midfrontal N2 in the self-related conditions, compared to the self-unrelated conditions, may indicate a smaller involvement of executive control in visual encoding and response execution^73,78,94,141^. The previous studies on self-prioritization usually interpreted such N2 effects in a similar fashion, as a decreased need for control of allocation of attentional or memory resources^27,33,53^.

#### The role of theta oscillations in visual processing

A strong theta-band activity in the posterior occipital cortex in response to an incoming stimulus is one of the electrophysiological signatures of receiving input in the visual system. Thus, it seems to reflect an aspect of basic visual processing. Unlike alpha-band oscillations, theta-band oscillations respond to increased processing- or task-demands only with an increase of amplitude or power—the effect termed as an event-related synchronization^142^. Thus, we may assume that the observed modulations of theta activity impact stimulus processing proportionally to the magnitude of these modulations. Thereby, the smaller increase of the stimulus-related theta activity in the self-related conditions indicates a smaller involvement of the processing “engines”. If the self-prioritization was obtained by attentional enhancement or amplification of self-related information, then we should observe an increase of the related theta activity.

How then to interpret the observed self-face-related theta effects? As proposed by Fries^132^, theta rhythm may serve resetting the gamma phase. More specifically, the function of theta activity is related to the governing attentional sampling of local gamma activity^143,144^. In the visual cortex, theta rhythm is engaged in top-down control loop of the gamma-band feedforward processing^132,145,146^. By resetting the gamma, theta oscillations control the input sent to the higher visual levels^144^. Weaker theta synchronization in the occipital areas may therefore indicate a less attentional or less controlled processing. Thus, it might be hypothesized that the smaller theta effects in visual areas reflect a more automatic processing of the stimuli. In other words, processing of self-related stimuli may be more automatized than processing of other information, at least in the visual areas. Further, the observed weaker stimulus-related theta ISPC in the self-relevant conditions suggests lower connectivity, therefore a lower inter-regional coupling^147^, which also may reflect a higher degree of automatization. In this line, Tacikowski, Freiburghaus and Ehrsson^6^ demonstrated that self-face recognition was associated with less cognitive interference than recognition of other faces. They have suggested that processing self-related information becomes automatized as a function of repetition: “the more frequently and consistently a mental representation is accessed, the lower its threshold of activation becomes”^6^ (p. 93). After sufficient training, certain representation activates in the presence of a trigger. This process becomes automatic, i.e., involuntary, preconscious, and performed with minimal cognitive effort or control. In conclusion, the present findings suggest that visual perception of one’s own face might be facilitated at the early sensory stages by means of a less effortful and more automatic processing of self-related information.

Interestingly, a recent macaque study proposed that less theta-rhythmic visual processing may indicate a more continuous processing of stimuli, which may subserve processing of behaviorally relevant information^148^. However, in that study, selective attention actually reduced, not increased, the strength of theta modulations in the visual cortex. This opens an alternative account of our results, as simply reflecting the effect of more focused attention on the self-related information. Still, such an effect of selective attention was not yet shown in humans. Hence, the issue seems opening a new line of research on the functional meaning of theta activity in self-referential visual processing.

#### Evoked and induced theta activity

The time–frequency-representation of task-related modulations of EEG power is a mix of evoked and induced activity (see e.g., Refs.^84,149^). The evoked activity is phase-aligned with the event onset, whereas the induced activity is the signal that is time-locked but not phase-locked to the event onset. Although it is not entirely clear what the two aspects of EEG signal reflect in terms of cognitive operations, it is often assumed that evoked activity may reflect transient non-sinusoidal oscillations that are event-like and time-intermittent (cf. Ref.^150^), whereas the presence of induced activity is usually interpreted as evidence for rhythmic, sinusoidal, and stimulus independent (or endogenous) oscillations^84,149^. Therefore, an issue to be addressed in future is the dissociation between the two types of activity and determining their functions in the processing of self-related stimuli.

**Figure 6:**
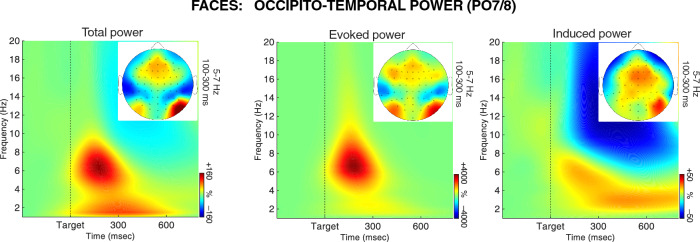
Face-related modulations of total (left panel), evoked (phase-locked; middle panel), and induced (non-phase-locked, right panel) EEG power from the occipito-temporal sites PO7 and PO8, along with topographies for the relevant time–frequency window (100–300 ms, 5–7 Hz). The time–frequency plots depict power spectra averaged over the Self Relevance conditions and PO7/8 sites.

In the current study, we did not have enough statistical power per participant to reliably examine this issue. Thus, we briefly report only an exploratory examination of a general difference between the two signals in the responses to faces. We isolated induced and evoked power by subtracting the ERP from the single-trial EEG signal, and then decomposed the phase-locked and non-phase-locked signals into their time–frequency representations (using the same method as for total power, see “Methods”; for details see Refs.^73,151^). The results showed that the early posterior (visual) stimulus-related theta-band activity (100–300 ms, 5–7 Hz) was present in both evoked and induced power (see Fig. 6). This suggests that the visual effects observed here in the total power analysis may include both types of neural oscillations. This is in agreement with the fact that the spectral analysis showed the self-face effects that were not detected in the ERPs.

### Task-relevance of self-stimuli

A factor that should also be considered when comparing visual self-effects from different studies is the specificity of task-demands, and particularly whether self-related information is task-related or task-irrelevant. Active and passive stimulus recognition are two different processes. Active recognition of one’s own face and its intentional (task-related) discrimination among other faces involve the top-down control and intentional conscious prioritization of self-relevant information^6,152^. Several studies on self-prioritization stressed the role of top-down attentional modulations in self-referential processing^131,153,154^, e.g., by showing the involvement of the lateral prefrontal areas and anterior cingulate cortex^155^. Self-prioritization appears to be an attentional effect especially from the perspective of research that failed to find self-effects when the self-related information was not task-relevant^156–158^. On the other hand, several other studies did find task-irrelevant self-referential effects^17,159^. Both the active and passive viewing of self-face activate brain regions associated with the face representation, e.g., the fusiform gyrus and supramarginal gyrus. Also, the anterior midline areas typically involved in self-related processing—the medial prefrontal cortex and posterior cingulate cortex—are often found active even if self-relevance discrimination was not explicitly required by the task^160–162^. In the present study, the task did not require active stimulus discrimination, and the results still showed several self-related modulations of the brain activity. Taken together, the results suggest that the self-face differentiation occurs automatically.

### Self-effects vs. familiarity effects

It is often raised as an issue whether self-face evokes different brain responses because it is a highly familiar stimulus^156,163^. Here, not only the familiar faces (famous and friend categories) evoked a different response than the self-face, but also the effects of familiar stimuli did not differ from the unknown stimuli. This adds evidence for the interpretation of the self-relevance effects, and brings us to the conclusion that personally relevant stimuli are more than just highly familiar stimuli (for similar results see Refs.^153,164,165^).

## Data Availability

The data supporting the reported findings are available from the corresponding author upon reasonable request.

## References

[1] Alexopoulos T, Muller D, Ric F, Marendaz C (2012). I, me, mine: Automatic attentional capture by self-related stimuli. Eur. J. Soc. Psychol..

[2] Moray N (1959). Attention in dichotic listening: Affective cues and the influence of instructions. Q. J. Exp. Psychol..

[3] Tacikowski P, Nowicka A (2010). Allocation of attention to self-name and self-face: An ERP study. Biol. Psychol..

[4] Wójcik MJ, Nowicka MM, Kotlewska I, Nowicka A (2018). Self-face captures, holds, and biases attention. Front. Psychol..

[5] Brédart S, Delchambre M, Laureys S (2006). One’s own face is hard to ignore. Q. J. Exp. Psychol..

[6] Tacikowski P, Freiburghaus T, Ehrsson HH (2017). Goal-directed processing of self-relevant information is associated with less cognitive interference than the processing of information about other people. J. Exp. Soc. Psychol..

[7] Zhao S, Uono S, Li C, Yoshimura S, Toichi M (2018). The influence of self-referential processing on attentional orienting in frontoparietal networks. Front. Hum. Neurosci..

[8] Chen Y (2014). Evidence for implicit self-positivity bias: An event-related brain potential study. Exp. Brain Res..

[9] Conway MA, Dewhurst SA (1995). The self and recollective experience. Appl. Cogn. Psychol..

[10] Heatherton TF (2006). Medial prefrontal activity differentiates self from close others. Soc. Cogn. Affect. Neurosci..

[11] Moran JM, Macrae CN, Heatherton TF, Wyland CL, Kelley WM (2006). Neuroanatomical evidence for distinct cognitive and affective components of self. J. Cogn. Neurosci..

[12] Symons CS, Johnson BT (1997). The self-reference effect in memory: A meta-analysis. Psychol. Bull..

[13] Tanguay AN (2018). The ERP correlates of self-knowledge: Are assessments of one’s past, present, and future traits closer to semantic or episodic memory?. Neuropsychologia.

[14] Cunningham SJ, Turk DJ (2017). Editorial: A review of self-processing biases in cognition. Q. J. Exp. Psychol..

[15] Legrand D, Ruby P (2009). What is self-specific? Theoretical investigation and critical review of neuroimaging results. Psychol. Rev..

[16] Tong F, Nakayama K (1999). Robust representations for faces: Evidence from visual search. J. Exp. Psychol. Hum. Percept. Perform..

[17] Bola M, Paź M, Doradzińska Ł, Nowicka A (2021). The self-face captures attention without consciousness: Evidence from the N2pc ERP component analysis. Psychophysiology.

[18] Gray HM, Ambady N, Lowenthal WT, Deldin P (2004). P300 as an index of attention to self-relevant stimuli. J. Exp. Soc. Psychol..

[19] Sedikides C, Gregg AP (2008). Self-enhancement food for thought. Perspect. Psychol. Sci..

[20] Caharel S, Courtay N, Bernard C, Lalonde R, Rebaï M (2005). Familiarity and emotional expression influence an early stage of face processing: An electrophysiological study. Brain Cogn..

[21] Caharel S (2007). The effects of familiarity and emotional expression on face processing examined by ERPs in patients with schizophrenia. Schizophr. Res..

[22] Geng H, Zhang S, Li Q, Tao R, Xu S (2012). Dissociations of subliminal and supraliminal self-face from other-face processing: Behavioral and ERP evidence. Neuropsychologia.

[23] Keyes H, Brady N, Reilly RB, Foxe JJ (2010). My face or yours? Event-related potential correlates of self-face processing. Brain Cogn..

[24] Zeman PM, Till BC, Livingston NJ, Tanaka JW, Driessen PF (2007). Independent component analysis and clustering improve signal-to-noise ratio for statistical analysis of event-related potentials. Clin. Neurophysiol..

[25] Hinojosa JA, Mercado F, Carretié L (2015). N170 sensitivity to facial expression: A meta-analysis. Neurosci. Biobehav. Rev..

[26] Olivares EI, Iglesias J, Saavedra C, Trujillo-Barreto NJ, Valdés-Sosa M (2015). Brain signals of face processing as revealed by event-related potentials. Behav. Neurol..

[27] Alzueta E, Melcón M, Poch C, Capilla A (2019). Is your own face more than a highly familiar face?. Biol. Psychol..

[28] Caharel S (2002). ERPs associated with familiarity and degree of familiarity during face recognition. Int. J. Neurosci..

[29] Cygan HB, Tacikowski P, Ostaszewski P, Chojnicka I, Nowicka A (2014). Neural correlates of own name and own face detection in autism spectrum disorder. PLoS One.

[30] Miyakoshi M, Kanayama N, Nomura M, Iidaka T, Ohira H (2008). ERP study of viewpoint-independence in familiar-face recognition. Int. J. Psychophysiol..

[31] Miyakoshi M, Kanayama N, Iidaka T, Ohira H (2010). EEG evidence of face-specific visual self-representation. Neuroimage.

[32] Pierce LJ (2011). The N250 brain potential to personally familiar and newly learned faces and objects. Front. Hum. Neurosci..

[33] Rubianes M (2020). Am I the same person across my life span? An event-related brain potentials study of the temporal perspective in self-identity. Psychophysiology.

[34] Scott LS, Luciana M, Wewerka S, Nelson CA (2005). Electrophysiological correlates of facial self-recognition in adults and children. Cogn. Brain Behav..

[35] Sui J, Zhu Y, Han S (2006). Self-face recognition in attended and unattended conditions: An event-related brain potential study. NeuroReport.

[36] Tanaka JW, Curran T, Porterfield AL, Collins D (2006). Activation of preexisting and acquired face representations: The N250 event-related potential as an index of face familiarity. J. Cogn. Neurosci..

[37] Żochowska A, Jakuszyk P, Nowicka MM, Nowicka A (2022). Are covered faces eye-catching for us? The impact of masks on attentional processing of self and other faces during the COVID-19 pandemic. Cortex.

[38] Caharel S, Rossion B (2021). The N170 is sensitive to long-term (personal) familiarity of a face identity. Neuroscience.

[39] Eick CM, Ambrus GG, Kovács G (2021). Inhibition of the occipital face area modulates the electrophysiological signals of face familiarity: A combined cTBS-EEG study. Cortex.

[40] Schweinberger SR, Calder AJ, Rhodes G, Johnson MH, Haxby JV (2011). Neurophysiological correlates of face recognition. Oxford Handbook of Face Perception.

[41] Wuttke SJ, Schweinberger SR (2019). The P200 predominantly reflects distance-to-norm in face space whereas the N250 reflects activation of identity-specific representations of known faces. Biol. Psychol..

[42] Tacikowski P, Cygan HB, Nowicka A (2014). Neural correlates of own and close-other’s name recognition: ERP evidence. Front. Hum. Neurosci..

[43] Miyakoshi M, Nomura M, Ohira H (2007). An ERP study on self-relevant object recognition. Brain Cogn..

[44] Liu J, Harris A, Kanwisher N (2002). Stages of processing in face perception: An MEG study. Nat. Neurosci..

[45] Debruille JB, Guillem F, Renault B (1998). ERPs and chronometry of face recognition: Following-up Seeck et al. and George et al. NeuroReport.

[46] Feng W, Martinez A, Pitts M, Luo YJ, Hillyard SA (2012). Spatial attention modulates early face processing. Neuropsychologia.

[47] Halit H, de Haan M, Johnson MH (2000). Modulation of event-related potentials by prototypical and atypical faces. NeuroReport.

[48] Rellecke J, Sommer W, Schacht A (2012). Does processing of emotional facial expressions depend on intention? Time-resolved evidence from event-related brain potentials. Biol. Psychol..

[49] Pourtois G, Grandjean D, Sander D, Vuilleumier P (2004). Electrophysiological correlates of rapid spatial orienting towards fearful faces. Cereb. Cortex.

[50] Jacques C, Caharel S (2022). The time course of categorical perception of facial expressions. Neuropsychologia.

[51] Deffke I (2007). MEG/EEG sources of the 170-ms response to faces are co-localized in the fusiform gyrus. Neuroimage.

[52] Rossion B, Joyce CA, Cottrell GW, Tarr MJ (2003). Early lateralization and orientation tuning for face, word, and object processing in the visual cortex. Neuroimage.

[53] Woźniak M, Kourtis D, Knoblich G (2018). Prioritization of arbitrary faces associated to self: An EEG study. PLoS One.

[54] Alzueta E, Kessel D, Capilla A (2021). The upside-down self: One’s own face recognition is affected by inversion. Psychophysiology.

[55] Cygan HB, Nowicka MM, Nowicka A (2022). Impaired attentional bias toward one’s own face in autism spectrum disorder: ERP evidence. Autism Res..

[56] Kotlewska I, Nowicka A (2015). Present self, past self and close-other: Event-related potential study of face and name detection. Biol. Psychol..

[57] Ninomiya H, Onitsuka T, Chen CH, Sato E, Tashiro N (1998). P300 in response to the subject’s own face. Psychiatry Clin. Neurosci..

[58] Żochowska A, Nowicka MM, Wójcik MJ, Nowicka A (2021). Self-face and emotional faces—Are they alike?. Soc. Cogn. Affect. Neurosci..

[59] Żochowska A, Jakuszyk P, Nowicka MM, Nowicka A (2022). The self and a close-other: Differences between processing of faces and newly acquired information. Cereb. Cortex.

[60] Fan W (2013). Electrophysiological correlation of the degree of self-reference effect. PLoS One.

[61] Fischler I, Jin YS, Boaz TL, Perry NW, Childers DG (1987). Brain potentials related to seeing one’s own name. Brain Lang..

[62] Nowicka A, Cygan HB, Tacikowski P, Ostaszewski P, Kuś R (2016). Name recognition in autism: EEG evidence of altered patterns of brain activity and connectivity. Mol. Autism.

[63] Niu G (2020). Behavioural and ERP evidence of the self-advantage of online self-relevant information. Sci. Rep..

[64] Kotlewska I, Nowicka A (2016). Present-self, past-self and the close-other: Neural correlates of assigning trait adjectives to oneself and others. Eur. J. Neurosci..

[65] Luo Y, Huang X, Chen Y, Jackson T, Wei D (2010). Negativity bias of the self across time: An event-related potentials study. Neurosci. Lett..

[66] Asanowicz D (2020). The response relevance of visual stimuli modulates the P3 component and the underlying sensorimotor network. Sci. Rep..

[67] Verleger R (2020). Effects of relevance and response frequency on P3b amplitudes: Review of findings and comparison of hypotheses about the process reflected by P3b. Psychophysiology.

[68] Verleger R, Asanowicz D, Werner L, Śmigasiewicz K (2015). Biased odds for heads or tails: Outcome-evoked P3 depends on frequencies of guesses. Psychophysiology.

[69] Twomey DM, Murphy PR, Kelly SP, O’Connell RG (2015). The classic P300 encodes a build-to-threshold decision variable. Eur. J. Neurosci..

[70] Polich J (2007). Updating P300: An integrative theory of P3a and P3b. Clin. Neurophysiol..

[71] Neuhaus AH (2010). Event-related potentials associated with Attention Network Test. Int. J. Psychophysiol..

[72] Asanowicz D, Wołoszyn K, Panek B, Wronka E (2019). On the locus of the effect of alerting on response conflict: An event-related EEG study with a speed-accuracy tradeoff manipulation. Biol. Psychol..

[73] Asanowicz D, Panek B, Kotlewska I (2021). Selection for action: The medial frontal cortex is an executive hub for stimulus and response selection. J. Cogn. Neurosci..

[74] Hopfinger JB, Mangun GR (1998). Reflexive attention modulates processing of visual stimuli in human extrastriate cortex. Psychol. Sci..

[75] Śmigasiewicz K, Asanowicz D, Westphal N, Verleger R (2015). Bias for the left visual field in rapid serial visual presentation: Effects of additional salient cues suggest a critical role of attention. J. Cogn. Neurosci..

[76] Zhao K (2009). Event-related potential correlates of the collective self-relevant effect. Neurosci. Lett..

[77] Vogel EK, Luck SJ (2000). The visual N1 component as an index of a discrimination process. Psychophysiology.

[78] Folstein JR, van Petten C (2008). Influence of cognitive control and mismatch on the N2 component of the ERP: A review. Psychophysiology.

[79] Cellerino A, Borghetti D, Sartucci F (2004). Sex differences in face gender recognition in humans. Brain Res. Bull..

[80] Haxby JV, Hoffman EA, Gobbini MI (2000). The distributed human neural system for face perception. Trends Cogn. Sci..

[81] Dien J (2009). A tale of two recognition systems: Implications of the fusiform face area and the visual word form area for lateralized object recognition models. Neuropsychologia.

[82] Dehaene S, Cohen L (2011). The unique role of the visual word form area in reading. Trends Cogn. Sci..

[83] Kanwisher N, Yovel G (2006). The fusiform face area: A cortical region specialized for the perception of faces. Philos. Trans. R. Soc. B Biol. Sci..

[84] Cohen MX (2014). Analyzing Neural Time Series Data: Theory and Practice.

[85] Buzsáki G (2006). Rhythms of the Brain.

[86] Zion-Golumbic E, Kutas M, Bentin S (2010). Neural dynamics associated with semantic and episodic memory for faces: Evidence from multiple frequency bands. J. Cogn. Neurosci..

[87] Başar E, Özgören M, Öniz A, Schmiedt C, Başar-Eroǧlu C (2007). Brain oscillations differentiate the picture of one’s own grandmother. Int. J. Psychophysiol..

[88] Bossi F (2020). Theta- and gamma-band activity discriminates face, body and object perception. Front. Hum. Neurosci..

[89] del Giudice R (2014). Oscillatory brain responses to own names uttered by unfamiliar and familiar voices. Brain Res..

[90] Höller Y (2011). EEG frequency analysis of responses to the own-name stimulus. Clin. Neurophysiol..

[91] Luck SJ, Gaspelin N (2017). How to get statistically significant effects in any ERP experiment (and why you shouldn’t). Psychophysiology.

[92] Rossion, B. & Jacques, C. The N170: Understanding the time course of face perception in the human brain. In *The Oxford Handbook of Event-Related Potential Components* 1–30. 10.1093/oxfordhb/9780195374148.013.0064 (2012).

[93] Verleger R, Smigasiewicz K, Möller F, Möller M (2011). Mechanisms underlying the left visual-field advantage in the dual stream RSVP task: Evidence from N2pc, P3, and distractor-evoked VEPs. Psychophysiology.

[94] Asanowicz D, Kotlewska I, Panek B (2022). Neural underpinnings of proactive and preemptive adjustments of action control. J. Cogn. Neurosci..

[95] Cohen MX (2017). MATLAB for Brain and Cognitive Scientists.

[96] Trujillo LT, Allen JJB (2007). Theta EEG dynamics of the error-related negativity. Clin. Neurophysiol..

[97] Cohen MX (2022). A tutorial on generalized eigendecomposition for denoising, contrast enhancement, and dimension reduction in multichannel electrophysiology. Neuroimage.

[98] Nikulin VV, Nolte G, Curio G (2011). A novel method for reliable and fast extraction of neuronal EEG/MEG oscillations on the basis of spatio-spectral decomposition. Neuroimage.

[99] Parra L, Sajda P (2003). Blind source separation via generalized eigenvalue decomposition. J. Mach. Learn. Res..

[100] de Cheveigné A, Parra LC (2014). Joint decorrelation, a versatile tool for multichannel data analysis. Neuroimage.

[101] Zuure MB, Hinkley LB, Tiesinga PHE, Nagarajan SS, Cohen MX (2020). Multiple midfrontal thetas revealed by source separation of simultaneous MEG and EEG. J. Neurosci..

[102] Cohen MX (2018). Using spatiotemporal source separation to identify prominent features in multichannel data without sinusoidal filters. Eur. J. Neurosci..

[103] Hayton JC, Allen DG, Scarpello V (2004). Factor retention decisions in exploratory factor analysis: A tutorial on parallel analysis. Organ Res. Methods.

[104] Haufe S (2014). On the interpretation of weight vectors of linear models in multivariate neuroimaging. Neuroimage.

[105] Cohen MX, Ridderinkhof KR, Haupt S, Elger CE, Fell J (2008). Medial frontal cortex and response conflict: Evidence from human intracranial EEG and medial frontal cortex lesion. Brain Res..

[106] Lachaux J, Rodriguez E, Martinerie J, Varela FJ (1999). Measuring phase synchrony in brain signals. Hum. Brain Mapp..

[107] Cohen MX (2015). Effects of time lag and frequency matching on phase-based connectivity. J. Neurosci. Methods.

[108] Srinivasan R, Winter WR, Ding J, Nunez PL (2007). EEG and MEG coherence: Measures of functional connectivity at distinct spatial scales of neocortical dynamics. J. Neurosci. Methods.

[109] Cohen MX (2015). Comparison of different spatial transformations applied to EEG data: A case study of error processing. Int. J. Psychophysiol..

[110] Northoff G (2017). Personal identity and cortical midline structure (CMS): Do temporal features of CMS neural activity transform into ‘self-continuity’?. Psychol. Inq..

[111] Northoff G (2006). Self-referential processing in our brain—A meta-analysis of imaging studies on the self. Neuroimage.

[112] Northoff G, Bermpohl F (2004). Cortical midline structures and the self. Trends Cogn. Sci..

[113] Wagner DD, Haxby JV, Heatherton TF (2012). The representation of self and person knowledge in the medial prefrontal cortex. Wiley Interdiscip. Rev. Cogn. Sci..

[114] Smith EE, Bel-Bahar TS, Kayser J (2022). A systematic data-driven approach to analyze sensor-level EEG connectivity: Identifying robust phase-synchronized network components using surface Laplacian with spectral-spatial PCA. Psychophysiology.

[115] Cavanagh JF, Cohen MX, Gable P, Miller M, Bernat E (2022). Frontal midline theta as a model specimen of cortical theta. The Oxford Handbook of EEG Frequency.

[116] Cavanagh JF, Frank MJ (2014). Frontal theta as a mechanism for cognitive control. Trends Cogn. Sci..

[117] Fries P (2005). A mechanism for cognitive dynamics: Neuronal communication through neuronal coherence. Trends Cogn. Sci..

[118] Cavanagh JF, Meyer A, Hajcak G (2017). Error-specific cognitive control alterations in generalized anxiety disorder. Biol. Psychiatry Cogn. Neurosci. Neuroimaging.

[119] McKewen M (2021). Dissociable theta networks underlie the switch and mixing costs during task switching. Hum. Brain Mapp..

[120] Bortolon C, Raffard S (2018). Self-face advantage over familiar and unfamiliar faces: A three-level meta-analytic approach. Psychon. Bull. Rev..

[121] Asanowicz D, Śmigasiewicz K, Verleger R (2013). Differences between visual hemifields in identifying rapidly presented target stimuli: Letters and digits, faces, and shapes. Front. Psychol..

[122] Borra D, Bossi F, Rivolta D, Magosso E (2023). Deep learning applied to EEG source-data reveals both ventral and dorsal visual stream involvement in holistic processing of social stimuli (123AD). Sci. Rep..

[123] Kanwisher N, McDermott J, Chun MM (1997). The fusiform face area: A module in human extrastriate cortex specialized for face perception. J. Neurosci..

[124] Haxby JV (1999). The effect of face inversion on activity in human neural systems for face and object perception. Neuron.

[125] Hamamé CM (2013). Dejerine’s reading area revisited with intracranial EEG. Neurology.

[126] Rossion B, Lochy A (2022). Is human face recognition lateralized to the right hemisphere due to neural competition with left-lateralized visual word recognition? A critical review. Brain Struct. Funct..

[127] Cohen L (2000). The visual word form area: Spatial and temporal characterization of an initial stage of reading in normal subjects and posterior split-brain patients. Brain.

[128] Berlad I, Pratt H (1995). P300 in response to the subject’s own name. Electroencephalogr. Clin. Neurophysiol./Evoked Potentials.

[129] D’Argembeau A (2010). Modulation of medial prefrontal and inferior parietal cortices when thinking about past, present, and future selves. Soc. Neurosci..

[130] Isoda M (2021). The role of the medial prefrontal cortex in moderating neural representations of self and other in primates. Annu. Rev. Neurosci..

[131] Humphreys GW, Sui J (2016). Attentional control and the self: The Self-Attention Network (SAN). Cogn. Neurosci..

[132] Fries P (2015). Rhythms for cognition: Communication through coherence. Neuron.

[133] Siegel M, Donner TH, Engel AK (2012). Spectral fingerprints of large-scale neuronal interactions. Nat. Rev. Neurosci..

[134] Kohn A (2020). Principles of corticocortical communication: Proposed schemes and design considerations. Trends Neurosci..

[135] Eger E, Jedynak A, Iwaki T, Skrandies W (2003). Rapid extraction of emotional expression: Evidence from evoked potential fields during brief presentation of face stimuli. Neuropsychologia.

[136] Batty M, Taylor MJ (2003). Early processing of the six basic facial emotional expressions. Cogn. Brain Res..

[137] Luo W, Feng W, He W, Wang NY, Luo YJ (2010). Three stages of facial expression processing: ERP study with rapid serial visual presentation. Neuroimage.

[138] Eimer M, Holmes A (2002). An ERP study on the time course of emotional face processing. NeuroReport.

[139] Eimer M, Holmes A (2007). Event-related brain potential correlates of emotional face processing. Neuropsychologia.

[140] Rellecke J, Sommer W, Schacht A (2013). Emotion effects on the n170: A question of reference?. Brain Topogr..

[141] Yeung N, Botvinick MM, Cohen JD (2004). The neural basis of error detection: Conflict monitoring and the error-related negativity. Psychol. Rev..

[142] Klimesch W (2012). Alpha-band oscillations, attention, and controlled access to stored information. Trends Cogn. Sci..

[143] Canolty RT (2006). High gamma power is phase-locked to theta oscillations in human neocortex. Science.

[144] Fiebelkorn IC, Kastner S (2020). Functional specialization in the attention network. Annu. Rev. Psychol..

[145] Bastos AM (2015). Visual areas exert feedforward and feedback influences through distinct frequency channels. Neuron.

[146] Fiebelkorn IC (2011). Ready, set, reset: Stimulus-locked periodicity in behavioral performance demonstrates the consequences of cross-sensory phase reset. J. Neurosci..

[147] Sel A (2019). Increasing and decreasing interregional brain coupling increases and decreases oscillatory activity in the human brain. Proc. Natl. Acad. Sci..

[148] Spyropoulos G, Bosman CA, Fries P (2018). A theta rhythm in macaque visual cortex and its attentional modulation. Proc. Natl. Acad. Sci. U.S.A..

[149] David O, Kilner JM, Friston KJ (2006). Mechanisms of evoked and induced responses in MEG/EEG. Neuroimage.

[150] Jones SR (2016). When brain rhythms aren’t ‘rhythmic’: Implication for their mechanisms and meaning. Curr. Opin. Neurobiol..

[151] Cohen MX, Donner TH (2013). Midfrontal conflict-related theta-band power reflects neural oscillations that predict behavior. J. Neurophysiol..

[152] Tacikowski P, Berger CC, Ehrsson HH (2017). Dissociating the neural basis of conceptual self-awareness from perceptual awareness and unaware self-processing. Cereb. Cortex.

[153] Sui J, He X, Humphreys GW (2012). Perceptual effects of social salience: Evidence from self-prioritization effects on perceptual matching. J. Exp. Psychol. Hum. Percept. Perform..

[154] Wozniak M, Hohwy J (2020). Stranger to my face: Top-down and bottom-up effects underlying prioritization of images of one’s face. PLoS One.

[155] Sugiura M (2000). Passive and active recognition of one’s own face. Neuroimage.

[156] Devue C, van der Stigchel S, Brédart S, Theeuwes J (2009). You do not find your own face faster; you just look at it longer. Cognition.

[157] Kawahara JI, Yamada Y (2004). Does one’s name attract visual attention?. Vis. Cogn..

[158] Gronau N, Cohen A, Ben-Shakhar G (2003). Dissociations of personally significant and task-relevant distractors inside and outside the focus of attention: A combined behavioral and psychophysiological study. J. Exp. Psychol. Gen..

[159] Doradzińska Ł (2020). Unconscious perception of one’s own name modulates amplitude of the P3B ERP component. Neuropsychologia.

[160] Moran JM, Heatherton TF, Kelley WM (2009). Modulation of cortical midline structures by implicit and explicit self-relevance evaluation. Soc. Neurosci..

[161] Rameson LT, Satpute AB, Lieberman MD (2010). The neural correlates of implicit and explicit self-relevant processing. Neuroimage.

[162] Sui J, Chechlacz M, Humphreys GW (2012). Dividing the self: Distinct neural substrates of task-based and automatic self-prioritization after brain damage. Cognition.

[163] Bortolon C, Lorieux S, Raffard S (2017). Self or familiar-face recognition advantage? New insight using ambient images. Q. J. Exp. Psychol..

[164] Sui J, Humphreys GW (2013). Self-referential processing is distinct from semantic elaboration: Evidence from long-term memory effects in a patient with amnesia and semantic impairments. Neuropsychologia.

[165] Sui J, Sun Y, Peng K, Humphreys GW (2014). The automatic and the expected self: Separating self- and familiarity biases effects by manipulating stimulus probability. Atten. Percept. Psychophys..

